# The Physiology of Hypnotism

**Published:** 1895-01

**Authors:** Alfred H. Porter

**Affiliations:** Philadelphia


### THE PHYSIOLOGY OF HYPNOTISM.[1]

BY ALFRED H. PORTER, D.D.S., PHILADELPHIA.

(Continued from Vol. XV., page 690.)

To summarize the foregoing, actual perception demands a phase of energy embracing both peripheral nervous elements of the retina of the eye and central nervous elements of the brain. Mental endeavor, although often concomitant with peripheral energies, demands the interdisposition of central energies alone, especially when it combines different phases to form new ideas. Having described simple perception as consisting of an undulating form of energy corresponding to that of the ethereal agitation in the environment and memory (which is the fundamental faculty of mind, for without it there would be nothing to deliberate, judge, or reason about) as the retention of this energy, we now proceed to the discussion of co-ordinated perception and consciousness and the arrest of the unison of energies effecting this by the hypnotic stimulus. In normal life these are ever varying with the continually changing environment. In the hypnotic condition a continuous strain exists, owing to the unceasing impact of waves on the retina representing the object regarded. This never-relaxing subtle but powerful stimulus antagonizing the anabolism of the tissues is eminently traumatic, acting as an insidious concussion no less potent because unseen, effecting the same results as visible impetus or pressure, and in the same way. Yet we must not forget that concussion, physical or mechanical, is but an exaggeration of normal sensation, that every sensation is an exciting stimulus whether the result of ethereal agitation of light and heat waves, affecting the retina and cuticle, aerial, acting on the tympanum, or of material pressure, visibly contactual with the sensory apparatus. These act precisely as the hypnotic stimulus, except that their effect is gradual on the higher centres of consciousness, and produce normal sleep after long-continued action. In their varied character allowing the recuperation, practically, the intermittent momentary sleep, of the tissues, lies the essential difference between them and the constant hypnotic stimulus. Normally, a katabolic disintegrating action continually goes on, restored to anabolic, high phases of energy by the metabolism of oxidation.

[1] An essay read before the Alumni Society of the Department of Dentistry, University of Pennsylvania.

If this affected all the cells of the retina at the same time, as it does in our hypnotic cases, no continuous consciousness could exist. In this fact lies the gist of the argument.

With an ever-varying environment at its maximum, as when a beautiful scene combines unity of design with diversity and graduation of hue and area, any individual cell subject to the wave-action corresponding to this will gradually exhibit less vibrant energies, until the oscillation is so low as to make use of the heat energies of the carbon and oxygen, and then rise again to its full period of vibration. The cell in the centre of the trough of the wave being subject to no exciting stimuli, will be recuperating, while that on the summit of the wave will be manifesting the high phases necessary to carry on the work of perception. As the wave proceeds to the brain, every cell in its course will exhibit a constantly-varying phase of energy, all manifesting the same phases, but at different times.

Every cell in the retina, according to the duration and size of the exciting environing wave, will exhibit alternately this downward, traumatic, excretory, dissociating wave, and a rising, associating, vitalizing positive wave. Virtually the rods dipping into pigment draw on metabolic resources to weigh as in a fine balance the various incoming waves. The fact that one cell can be among the low heat energies recuperating chemically and physically, while another is exhibiting high phases, secures the continuity of perception.

The clue to this possibility of the energies of the cell is that the environment is graduated and so excites lower differential phases until the lowest metabolic phase is reached, then it excites higher phases until the highest anabolic phase is reached. This only happens in a perfectly normal state, the energies of the cell being enabled to respond to this form of excitement by the maintenance of an equilibrium. This scheme secures two important things. In order to perceive a fine line or delicate shade in the landscape, a definite tension of the cell is needed, and is effected by a chemical association responding to the specific wave. But with an ever-changing environment such an association may not be needed for more than a second. It is eliminated by the downward negative wave. A new wave continually stimulates, requiring a new association, and this is effected by the upward positive wave. The rising positive waves not only must respond to a definite color and size, but several cells conjointly must excite a large cell in the molecular layer with which they are connected by nerves. The reason of this is that in the environment we find an intricate design composed of simple parts. The rods and cones respond to the different parts, and conjointly form a wave to represent the complex design. The large cells receive this compound wave, and so represent the contour or complex object perceived, for this combining power is their special function. On a larger scale the ganglia of the brain, gray cells, and pigment layers are foci of differentiated waves of energy, representing what has been seen, heard, and felt, coursing as electrical currents along the nerves, and held static by the magnetism of the cells, thus effecting co-ordinated apprehension, judgment, and reason. In this case the energies of the large cell or ganglion may not represent a composite design directly perceived in the landscape, but the conception of an artist or inventor. He may deem the design the creation of his imaginative genius and entirely original. It may be so in the light that his was the first brain in which the combination occurred, the first brain with the spider cell corresponding to the idea. But the elements of any intellectual or emotional association, whether that be picture, invention, poem, or simple plan of daily work, are represented by the energies of single cells, developed from the neuroglia of the brain in response to external excitation primarily. Our very consciousness of our surroundings, which departs at the end of the day's activities, is dependent on energy of large ganglia contributed by single cells throughout the night.

The rising constructive wave furnished by these is of special interest. For while in the visual centre a branched cell is an anatomical representation of the main branches of an external truncated object, the compound wave formed of myriads of smaller ones is the physiological representation of our conception of the outer world of hue and configuration in all its detail,—it is the electrical vibratory picture before referred to. The single cell acts as a di-electric; it is kept in a state of tension by the equipoise of opposite electricities, enabling it to respond to a stimulus, and excite contiguous nerves just to the extent of that stimulus for co-ordinating purposes. But if the tissue through malnutrition or irritation is not strong enough to bear the strain of this equipoise and separate the electricities for the directive purpose described, the union of these will cause a current to circulate through the cell. Any stimulus of whatever phase, however slight, will impress itself on the energies disproportionately, and the full current will exhibit this phase.

No combining power is possessed now by the various cells, but all clearness of outline and definiteness of configuration is lost in the general exaltation. A confused figure will apparently be seen in the landscape, suffused with light, a normal optical illusion.

We have been describing the effect of the ever-varying normal environment, and found in it destructive and constructive elements. We can picture the effect of a constant hypnotic stimulus, allowing of no rising constructive waves, but creating extreme tension by an all-prevailing negative wave keeping down a strong positive wave. According to the strength of the cell enabling it to stand this strain of opposed electricities will a discharge take place.

If the stimulus be not too severe, and the tissues normal, this will be discharged by the metabolic agencies, and normal sleep will ensue. Otherwise an explosion will occur traumatically, a current will circulate through the cell and involve the retina and the whole nervous system. This hyperæsthetic condition is the essential characteristic of hypnosis, differentiating it from ordinary sleep, rendering suggestion and control possible from external sources. If the term "hypnosis" means wakeful, somnambulistic sleep, the condition under consideration is rightly designated. But it means the reverse. It designates the anæsthetic condition of the nerves when the current, after circulating with increasing vehemence, and exhibiting the traumatic negative phase, is discharged by metabolic exchange. In this condition the cell will not even respond to the irritating, irregular action of tense waves denoting pain, for it is depleted of its idiostatic responsive forms of energy. This paralyzed condition is the effect of physical and chemical narcotic alike, after intense stimulation.

The characteristic of the normal cell as we described it is that its energies rise and fall, and exhibit exactly the same amount of energy as the exciting source, and thus maintain an equipoise of energies and a constant temperature.

The abnormal cell exhibits energies out of proportion to the exciting source, instead of retaining its energy and using it to restrain contiguous energy (the *raison d'être* of deliberation and self-control), it gives up its energies to form a current to circulate through the brain, inhibiting consciousness and all the faculties of the mind.

The very energy that accelerates the unrestrained passions is formed of the energy which normally governs them.

The circulating energies explain the phenomena of the exalted memory, and delusions characteristic of hypnotic states. Not only will a slight noise be magnified and seem deafening, or a point of light seem to swell in size, but the central energies of the brain itself furnish phases according to the ideas they represent. The free current energy will be impressed with that of whatever cell it comes into contact with, directed by the simple suggestion of the operator through the medium of the word centre.

This is the hyperæsthetic and hyperactive condition of all forms of amentia, whether the exciting source be the atomic energies of chemical hypnotics, malnutrition, overwork, or the hypnotic stimulus of a wave of light. That dominance of general, central activities allowing the accomplishment of regulated, governed, co-ordinated actions and thoughts, is replaced by the dominance of current activities surging through the nervous elements, the lower forms of mental activity prevail, delirium and resurrection of the past experience of life, auræ, convulsions, and unrestrained exhibitions of conduct generally.

(To be continued.)

